# Reduction of microglial activity in a model of multiple sclerosis by dipyridamole

**DOI:** 10.1186/1742-2094-10-89

**Published:** 2013-07-18

**Authors:** Scott Sloka, Luanne M Metz, Walter Hader, Yves Starreveld, V Wee Yong

**Affiliations:** 1 Hotchkiss Brain Institute and the Department of Clinical Neurosciences, University of Calgary, 3330 Hospital Drive, Calgary, AB T2N 4N1, Canada

**Keywords:** Cytokine, EAE, Inhibitor, Macrophage, Microglia

## Abstract

**Background:**

Despite extensive and persistent activation of microglia in multiple sclerosis (MS), microglia inhibitors have not yet been identified for treatment of the disorder. We sought to identify medications already in clinical use that could inhibit the activation of microglia. On the basis of the reported inhibitory effects of dipyridamole on phosphodiesterase activity that result in the production of various anti-inflammatory outcomes, we selected it for study. Dipyridamole is used clinically for secondary prevention in stroke. In this study, dipyridamole was examined using microglia in culture and in the mouse model of MS, experimental autoimmune encephalomyelitis (EAE).

**Results:**

We found that dipyridamole attenuated the elevation of several cytokines and chemokines in human microglia caused by Toll-like receptor stimulation. Morphological characteristics of activated microglia in culture were also normalized by dipyridamole. In mice, dipyridamole decreased the clinical severity of EAE and reduced microglial activity and other histological indices of EAE in the spinal cord.

**Conclusions:**

Dipyridamole is an inhibitor of microglia activation and may have a role in MS and other neurological conditions to attenuate microglial activity.

## Background

Multiple sclerosis (MS) is a chronic inflammatory demyelinating disease of the central nervous system (CNS) characterized by axonal and neuronal injury and loss. A predominant group of inflammatory cells in active plaques are highly activated phagocytic macrophages and microglia [1-3]. Because activated microglia cannot be reliably differentiated from blood-derived macrophages that have infiltrated the CNS, these cells are often collectively referred to as macrophages/microglia. Activated macrophages/microglia persist through secondary progressive MS [2].

The chronic presence of activated macrophage/microglia in MS is likely undesirable for several reasons. First, a strong correlation is observed between macrophage/microglia activity and both acute axonal injury [4,5] and loss of oligodendrocytes [6]. Second, medium conditioned by microglia kills oligodendrocytes in culture [7]; in MS lesions, microglia in the process of stripping myelin can be found [8]. Third, there is persistent activation of microglia in relapsing-remitting experimental autoimmune encephalomyelitis (EAE) even after CD4+ number wanes [9]. Finally, reducing macrophage/microglia activity with clodronate liposomes or genetic deletion alleviates disease activity in EAE [10,11].

Immunomodulators currently used in MS do not target microglial activity directly, and therefore the persistent macrophage/microglia activation within the CNS parenchyma remains a therapeutic gap. The tetracycline derivative, minocycline, has microglia inactivating properties in addition to other actions (reviewed in [12,13]) and is currently in a phase III trial in early MS (ClinicalTrials.gov identifier NCT00666887) following upon encouraging results in small trials in relapsing-remitting MS [14,15]. We have sought other approved medications with microglia-inhibitory activity for their potential utility in MS.

Dipyridamole has multiple known physiological effects [16]. It is primarily recognized as a platelet inhibitor and is used in combination with low-dose aspirin for secondary prevention in stroke [17]. Its antiplatelet mechanism is due in part to inhibition of adenosine uptake into platelets. The increased extracellular concentration of adenosine results in vasodilation. The combination of platelet inhibition and vasodilation promotes improved tissue perfusion. Dipyridamole also inhibits adenosine deaminase, which normally breaks down adenosine into inosine, further increasing concentrations of extracellular adenosine. Adenosine also has both neuroprotective and anti-inflammatory roles [18,19]. Dipyridamole is a phosphodiesterase inhibitor, which results in various anti-inflammatory outcomes [20,21]. Indeed, as a result of the phosphodiesterase-inhibitory activity, dipyridamole reduces the level of tumor necrosis factor α (TNF-α) produced by activated rodent microglia in culture [22,23]. TNF-α is an important proinflammatory factor at sites of injury and therefore is an attractive target for immunomodulatory therapy [24]. Recently, the anti-inflammatory roles of dipyridamole were utilized in a rat model of arthritis, where the results showed that prophylactic treatment reduced the arthritis-associated pathology [25].

To the best of our knowledge, there have been no published reports to date of the effect of dipyridamole in MS or EAE. Given that current MS therapies do not target microglial activity to any significant extent and that dipyridamole is an approved medication, we tested the utility of dipyridamole as an inhibitor of microglial responses. We have found that dipyridamole affects various aspects of microglial activity in culture and that dipyridamole-treated mice had reduced clinical and histological outcomes of EAE corresponding to reduced macrophage/microglia activity.

## Results

### Microglia are not killed by dipyridamole

**Figure 1:**
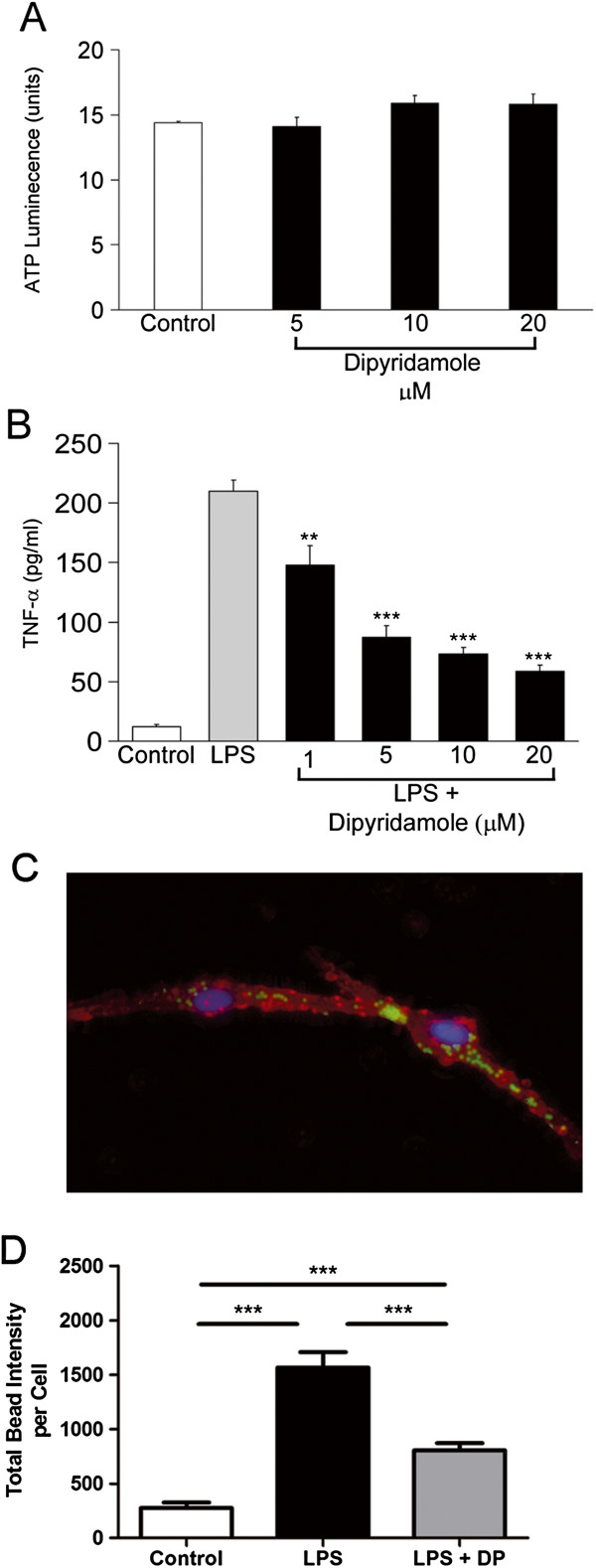
**Nontoxic concentrations of dipyridamole reduce TNF-α levels and phagocytic capacity of activated microglia. (A) **An ATP luminescence assay demonstrates that dipyridamole does not adversely affect viability to at least 20 μM. **(B)** TNF-α increases due to the addition of lipopolysaccharide (LPS) to microglia are attenuated by dipyridamole in a concentration-dependent fashion. **P < 0.01, ***P < 0.001 compared to LPS group. All values are means ± SEM (n = 4 wells per column) and were reproduced in three separate experiments. **(C) **The phagocytic capacity of microglia (Iba-1 label, red) is demonstrated by their ingestion of microfluorospheres (green) that were added to the culture medium. Note that this is an example of beads captured in a cell and that the quantitation in panel **(D)** depicts the results captured from all the cells in each well. Original magnification, ×200. **(D) **The total increase in bead intensity per cell due to LPS activation is attenuated by 10 μM dipyridamole (DP). Mean ± SEM of four wells per column. ***P < 0.001.

We tested dipyridamole on the viability of microglia and found that concentrations that can be achieved in humans after oral consumption (1 to 20 μM) [26] did not reduce cell viability (Figure 1A). These concentrations of dipyridamole were used for the remainder of the experiments.

Microglia from adult human brain were incubated with lipopolysaccharide (LPS) or LPS with dipyridamole, and the conditioned medium was harvested after 24 h. LPS increased TNF-α, but this effect was attenuated by concomitant treatment with dipyridamole (Figure 1B). Similar results (data not shown) were observed for fetal human microglia and for monocytes from the peripheral blood of adult volunteers.

We performed a phagocytosis assay to measure the function of microglia. Microglia were treated for 48 h with LPS with or without dipyridamole. Microfluorospheres were then added to the culture. Labeling of microglia with anti-Iba1 indicated ingestion of the fluorospheres (Figure 1C). Uptake of the fluorospheres was quantified by determining the total intensity of the fluorospheres within the microglia using algorithms developed in MATLAB (MathWorks, Natick, MA, USA) (Figure 1D). LPS increased the capacity of microglia to phagocytose the fluorospheres, but this effect was attenuated by the presence of dipyridamole (Figure 1D).

**Figure 2:**
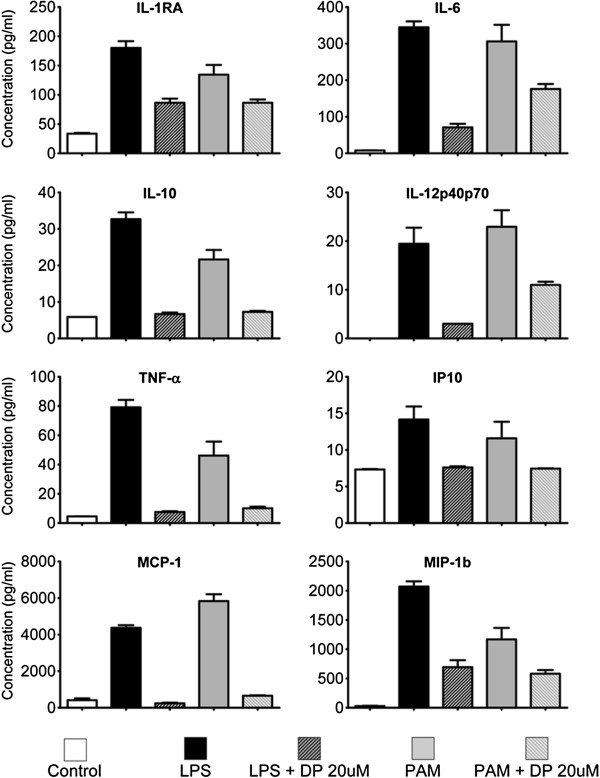
**The increase of multiple cytokines and chemokines following LPS or PAM activation of microglia is attenuated by dipyridamole. **25-cytokine multiplex ELISA permitted measurement of granulocyte-macrophage colony-stimulating factor, interferon α (IFN-α), IFN-γ, interleukin 1ra (IL-1ra), IL-1β, IL-2, IL-2R, IL-4, IL-5, IL-6, IL-7, IL-8, IL-10, IL-12, IL-13, IL-15, IL-17, IFN-γ-inducible protein 10 (IP-10), monocyte chemoattractant protein 1 (MCP-1), monokine induced by IFN-γ (MIG), macrophage inflammatory protein 1α (MIP-1α), MIP-1β, RANTES (regulated on activation, normal T cell expressed and secreted), eotaxin and TNF-α in the cell culture supernatant. LPS and Pam increased the levels of all inflammatory molecules, and all were attenuated by dipyridamole (20 μM). These data were reproduced in a second microglia culture. Only eight of the molecules are displayed.

Next, conditioned medium from microglia cultures was subjected to a 25-cytokine multiplex assay to determine the spectrum of molecules affected by dipyridamole. Selected molecules are displayed in Figure 2, but all results demonstrated a similar trend. Treatment with LPS or a Toll-like receptor 2 agonist, Pam, increased the secretion of proinflammatory molecules, but in all cases this effect was suppressed by dipyridamole (Figure 2).

### Dipyridamole normalizes cell morphology

**Figure 3:**
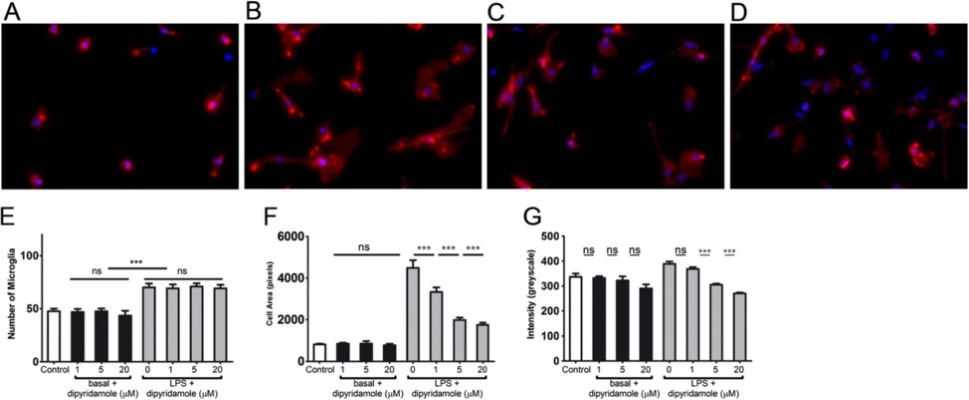
**Transformation of activated microglia to an amoeboid form was reduced when microglia under basal culture conditions or stimulated with 100 ng/ml LPS were exposed to dipyridamole for 2 days. (A) **through **(D) **CD14 staining. **(A)** Basal control microglia. **(B) **LPS alone. **(C) **LPS + 5 μM dipyridamole. **(D) **LPS + 20 μM dipyridamole. Original magnification, ×200. **(E)** Cell counts of microglia in LPS-stimulated or basal conditions were unaltered by dipyridamole. In contrast, the increase of cell area **(F) **and CD14 intensity **(G)** due to LPS activation was blocked by dipyridamole. This effect was dose-dependent. Dipyridamole did not alter these features in unstimulated basal microglia cultures. ***P < 0.001 compared to the respective controls. Values are means ± SEM of quadruplicate cultures. NS = not significant.

We next compared the morphological characteristics of control (untreated) microglia with those exposed to LPS, with and without dipyridamole, to evaluate microglial activation. Two days after treatment, cells were fixed and labeled for the monocytoid marker CD14 and for nuclei using Hoechst dye. Figure 3 emphasizes the high purity of the microglia cultures in that virtually all cells marked by the Hoechst dye were CD14-immunoreactive. Control microglia were small (Figure 3A), but those activated with LPS were morphologically larger (Figure 3B). This LPS-induced change appeared qualitatively to be prevented by dipyridamole (Figure 3C and 3D).

We quantitated the cellular characteristics using ImageXpress^MICRO^ (IXM, Molecular Devices, Sunnyvale, CA), a high-throughput cellular imaging system. Images from 96-well plates were acquired on the IXM, which consists of imaging hardware controlled by MetaXpress, the image analysis software. Images were also quantitated by MATLAB software. The addition of LPS increased cell counts compared with controls (Figure 3E) (*P* = 0.0008), a result we have consistently seen in other experiments. The administration of dipyridamole at increasing concentrations did not influence the basal or LPS-induced increase in cell numbers, which further substantiates that dipyridamole does not affect microglial viability. However, the average microglial cell area that was increased by LPS (*P* = 0.0007) was normalized by increasing concentrations of dipyridamole (Figure 3E). Although unaltered by dipyridamole in the unactivated state (*P* > 0.05), the average intensity of CD14 was reduced in activated microglia cultures by dipyridamole (Figure 3F).

In summary, dipyridamole does not alter the cell number in LPS-treated microglia cultures, but it maintains morphological alteration, cell area and CD14 intensity caused by LPS activation at near-normal levels. Because the effects of dipyridamole are observed only in LPS-treated and not basal cultures, the results suggest that dipyridamole is an inhibitor of microglia only when microglia become activated.

### Dipyridamole treatment reduces clinical and histological scores in EAE correspondent with decreased microglia activity

**Figure 4:**
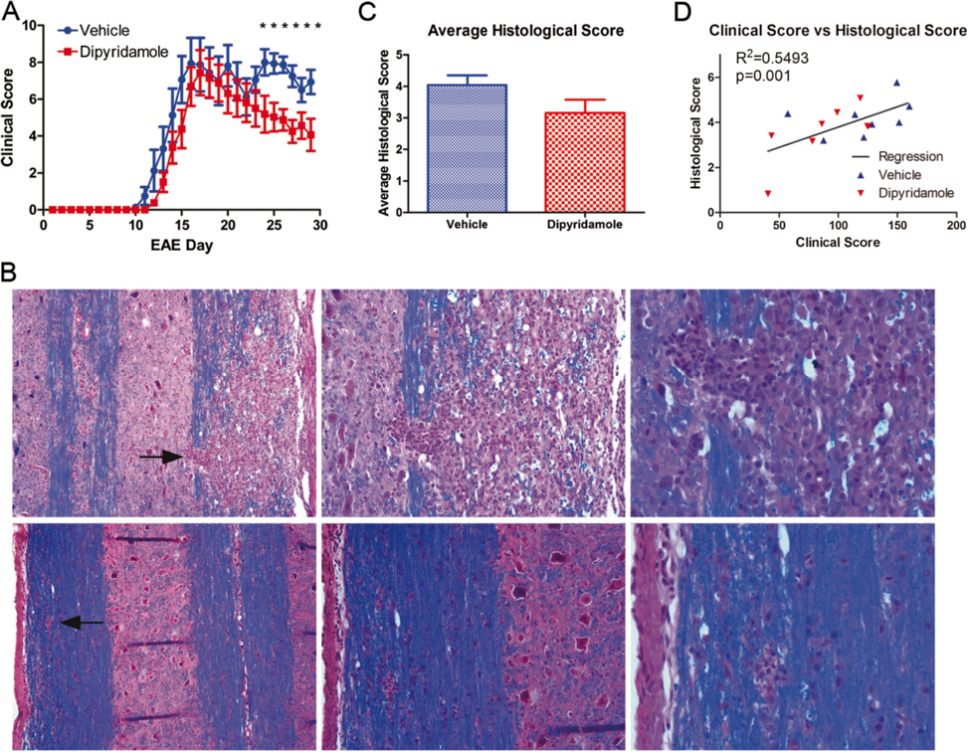
**Dipyridamole reduces clinical and histological scores of EAE. (A) **Clinical scores were reduced by 100 mg/kg dipyridamole (*P < 0.05) (*n *= 8 per group, mean ± SEM). **(B) **Hematoxylin and eosin and Luxol fast blue histological staining show multiple cellular infiltrates and demyelination in the vehicle-treated EAE group (top three panels at ×100, ×200 and ×400 original magnification, respectively, from left to right), which were reduced by dipyridamole (bottom three panels at ×100, ×200 and ×400 original magnification, respectively, from left to right). Arrows indicate the infiltrates that are highlighted in the higher-magnification photomicrographs. **(C) **Histological scores in dipyridamole-treated animals are reduced compared with vehicle-treated controls. **(D) **Clinical scores (represented as the sum of daily scores for each mouse over the entire experiment) correlated with histological scores (*P *< 0.001).

In mice treated daily with 100 mg/kg dipyridamole orally from day 7 postimmunization, the initial onset and peak of clinical signs were not altered, but continued treatment reduced clinical severity in the chronic phase of disease (Figure 4A). There were more and larger foci of inflammation and demyelination in the spinal cords of the vehicle-treated group compared to the dipyridamole-treated group (Figure 4B). Histological scores encompassing the extent of both inflammation and demyelination [27] were determined across multiple spinal cord specimens per mouse by a blinded evaluator. The histological score in vehicle-treated EAE mice was reduced by dipyridamole treatment (Figure 4C). Across both the dipyridamole- and vehicle-treated mice, there was a moderate correlation between the extent of histology and clinical score (Figure 4D).

**Figure 5:**
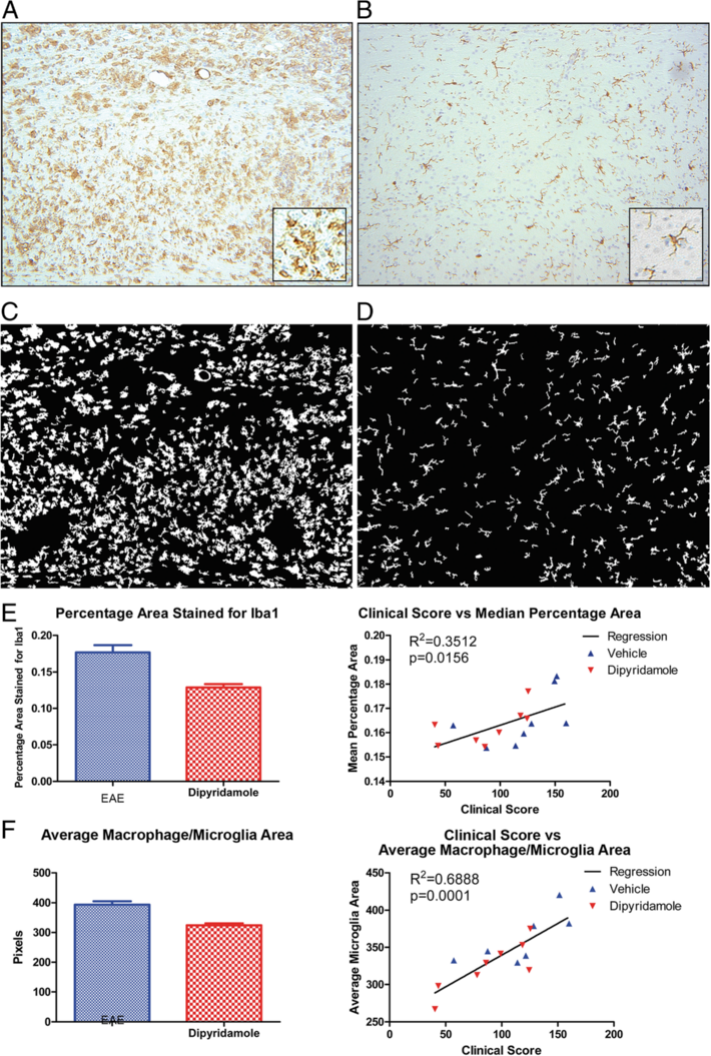
**Macrophage/microglia reactivity in EAE is reduced by dipyridamole. **Iba1 staining of thoracic cord shows extensive representation of macrophage/microglia in the vehicle group **(A) **upon killing of the mice (at termination of the experiment in Figure 4) compared to that with dipyridamole treatment **(B) **(×100 original magnification). The insets in **(A) **and **(B) **are high-magnification photomicrographs (×400 original magnification) that display the amoeboid versus ramified morphology, respectively, of cells. **(C) **and **(D)** Thresholded Iba1 staining of **(A) **and **(B)**, respectively, to allow the quantitation displayed in **(E) **and **(F)**. The percentage of spinal cord section occupied by Iba1 staining **(E) **and the average macrophage/microglia area **(F) **are reduced by dipyridamole compared to the EAE-vehicle group. These features correlate with the clinical scores (sum of daily scores of individual mice).

Nine sections per spinal cord were evaluated for macrophage/microglia reactivity following Iba1 staining. There was extensive Iba1 staining in sections from vehicle-treated EAE mice (Figure 5A). This was corroborated by the larger and amoeboid size of Iba1-stained cells. Spinal cord sections from dipyridamole-treated mice had predominantly ramified-shaped, Iba1-stained cells, indicative of unactivated microglia (Figure 5B).

To quantitate macrophage/microglia reactivity, digital images of the Iba1-stained sections were analyzed using the morphological image analysis functions in MATLAB (Figure 5C and 5D). These were compared with the clinical score. The percentage area of sections stained for Iba (Figure 5E), as well as the average microglial cell area (Figure 5F), was reduced in the dipyridamole-treated sections compared to vehicle-treated sections. The clinical score correlated with the microglia characteristics (Figure 5E and 5F), supporting a therapeutic effect of dipyridamole in EAE partly by a reduction of microglia reactivity.

### Impact of dose and time of administration on the therapeutic effect of dipyridamole

**Figure 6:**
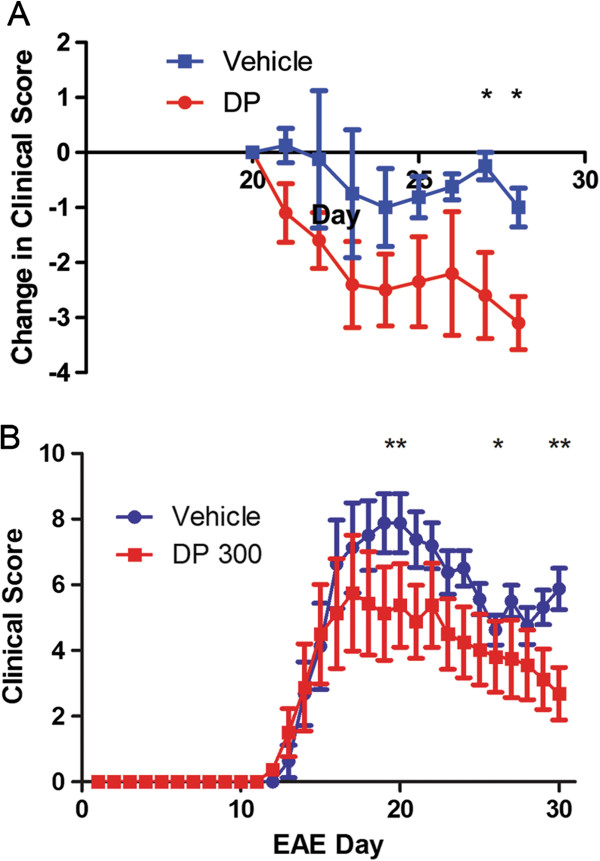
**Impact of dipyridamole dose and the time of treatment initiation on EAE. (A)** The clinical scores favored the dipyridamole-treated animals (*n *= 5 per group, mean ± SEM) after 8 d of treatment when initiation of treatment (100 mg/kg) was delayed until day 20 of EAE. **(B) **The clinical score in animals treated on day 7 of EAE induction with either 200 mg/kg (not shown) or 300 mg/kg dipyridamole is reduced at both the peak of disease and postpeak (*n *= 8 per group, mean ± SEM). *P < 0.05, **P < 0.01.

When dipyridamole was initiated after peak disease, dipyridamole-treated mice had greater resolution of clinical disease than vehicle-treated controls (Figure 6). When either 200 mg/kg or 300 mg/kg of oral dipyridamole was administered prior to onset of clinical symptoms (day 7 after myelin oligodendrocyte glycoprotein (MOG) immuni-zation), the increased dosage significantly reduced the severity of peak EAE disease (Figure 6B). The subsequent course was also reduced compared to vehicle treatment. There was no significant difference between the scores of the mice treated with 200 vs 300 mg/kg dipyridamole (data not shown).

## Discussion

Microglia populate the brain during early fetal development [28] and are quite plastic [29,30]. Unactivated microglia in postpartum life are ramified in morphology, but they transition to an amoeboid form in response to a variety of insults to the nervous system [31]. Unactivated microglia have many processes projecting from the cell, but, when activated, these processes retract and thicken and the microglia take on a more amoeboid, macrophage-like appearance. During CNS injury, monocytes infiltrate the CNS parenchyma and become amoeboid macrophages [29-32]. Thus, it becomes difficult to differentiate the activated microglia from infiltrated macrophages, and they are referred to collectively as *macrophages/microglia*. Recent studies indicate that the ramified microglia are not quiescent, but continually survey their microenvironment with extremely motile processes and protrusions [33].

In their activated, amoeboid form, microglia have the capacity to upregulate and release a myriad of secretory products that can contribute to defense but may also damage or kill neurons [29-31,34,35]. These products promote neuroinflammation, and it has been postulated that inflammatory mediators (cytokines, chemokines and free radicals) released from activated microglia contribute to the damage of neural cells. Nonetheless, in some contexts, microglia have neuroprotective properties [36,37], and the balance between neurotoxicity and neuroprotection may be a delicate one.

Blocking the activation of microglia reduces the detrimental effects of autoimmune-specific neuroinflammation. EAE is reduced in the CD40-depleted CNS [38] and in mice where activated microglia/macrophages are deleted through genetic manipulation [11]. Given these observations and that activated macrophage/microglia are present in high and persistent amounts in the CNS of patients with MS [1,2], a medication that suppresses microglia or normalizes its activity in MS could be a useful therapy. However, there is a dearth of such treatments available. One candidate therapy, minocycline, has microglia-inactivating and other immunomodulatory properties [13] and is currently in a phase III trial in early MS.

We now describe that dipyridamole is a candidate medication to inhibit microglial activation in MS. We have demonstrated that the activation of microglia in culture is normalized by dipyridamole treatment. In mice, dipyridamole inhibits EAE clinically and histologically, and this effect is correlated with reduced signs of macrophage/microglia activation examined by Iba1 immunohistochemistry. Other features of dipyridamole further support its potential utility in MS, and, because it is already in long-term use for stroke prevention, its potential chronic use in patients with MS has a clinical precedent. Dipyridamole also has antioxidant effects [39], and it inhibits the expression of matrix metalloproteinase 9 (MMP-9) [40,41]. These molecules are thought to contribute to MS pathogenesis. Dipyridamole has also been shown to suppress the release of soluble CD40 ligand [42], an effect that can reduce antigen presentation and inflammatory cascades.

### Summary of the effects of dipyridamole on microglia and experimental autoimmune encephalomyelitis

Reduces the morphological changes of microglia upon their activation.

Attenuates a spectrum of cytokines and chemokines secreted by activated microglia.

Prevents the activation of microglia, but not their transition from one subtype to another.

Decreases the severity of EAE when given prior to the onset of clinical signs.

Reduces the severity of chronic EAE when initiated after clinical signs are apparent.

Prevents the activation of microglia that occurs in EAE.

In recent years, macrophages have been subdivided into M1 and M2 phenotypes [43-45]. M1 is considered proinflammatory, produces proinflammatory cytokines such as TNF-α and is a strong expressor of C-C chemokine receptor type 2 (CCR2). M2 is thought to be regulatory/anti-inflammatory, to secrete regulatory cytokines such as IL-10, and express prominently C-X3-C motif chemokine receptor 1 (CX3CR1). Our data suggest that dipyridamole reduces activated microglia to a normal state, rather than being a regulator of the differentiation of cells into those that are M1 or M2. For example, both TNF-α and IL-10 are decreased in activated microglia by dipyridamole (Figure 2).

It is not known how much dipyridamole enters into the CNS after oral administration in humans or mice. However, given its highly lipophilic nature, a feature that favors entry into the CNS, it is anticipated that dipyridamole crosses the blood-brain barrier to affect microglia within. That said, we cannot exclude the possibility that some of the benefits of dipyridamole in EAE could be accounted for by peripheral mechanisms, such as the reduction of peripheral blood monocyte activity and hence their migration into the CNS to become macrophages. That dipyridamole did not affect the initial rise of clinical signs in a dose that attenuated final clinical and histological outcomes, however, suggests that the peripheral mechanisms that give rise to the onset of clinical signs do not need to be affected by dipyridamole. Furthermore, because treatment with dipyridamole was initiated on day 7, suppression of the initiating peripheral immunological mechanisms could not be explained by treatment with dipyridamole.

Platelets have been proposed to have roles in MS [46]. In this regard, platelets produce a variety of MMP members that are implicated in MS, and platelets also express several chemokine receptors that can respond to chemokines known to be elevated in MS [46]. There appears to be chronic platelet activation in MS, as evidenced by elevation of platelet microparticles and the platelet activation marker CD62L [47]. Recently, Langer *et al*. [48] found platelets in MS and EAE brain lesions and demonstrated that the depletion of platelets in mice ameliorated EAE disease course. Thus, the extent to which the antiplatelet activity [17] of dipyridamole helps account for its effectiveness in EAE remains to be determined. The results of our present study extend the spectrum of activity of dipyridamole to include microglia inhibition.

## Conclusions

In summary, we have found that dipyridamole normalizes macrophage/microglia activity and reduces the severity of EAE. Dipyridamole may counter the persistent activation of macrophage/microglia in MS, a feature that is not adequately treated by current medications proven to be effective in MS. One should be cautious when extrapolating from the current animal studies to humans, however, and dipyridamole may not work in MS. Given that microglial activation is a feature of many neurological conditions [48], dipyridamole should be tested across a spectrum of disorders where it is pertinent to normalize the activity of activated microglia.

## Methods

### Preparation and treatment of human microglia

Microglia of over 95% purity were isolated from the brains of adult humans undergoing resection to treat intractable epilepsy or from the brains of human fetuses of 10 to 18 wk gestational age, as previously described [49,50]. The use of these specimens was approved by the University of Calgary Research Ethics Board. Cells were plated in 96-well flat-bottomed black plates (BD Pharmingen, San Jose, CA, USA) at a density of 10,000 per well. The feeding medium used was minimum essential medium (MEM) supplemented with 10% fetal bovine serum, 1% penicillin/streptomycin, 0.1% dextrose, 1× nonessential amino acids, 10 μM glutamine and 1 mM sodium pyruvate (called *complete MEM*).

Where indicated, adherent cells were treated with 100 ng/ml of the Toll-like receptor 4 agonist LPS (100 ng/ml); the Toll-like receptor 2 agonist Pam; a synthetic tripalmitoylated cysteine-, serine- and lysine-containing peptide; and with varying concentrations of dipyridamole. All treatments with dipyridamole were done 30 min prior to the addition of LPS or Pam. The pretreatment period was necessary because LPS and Pam are potent activators that trigger signaling cascades minutes after engagement of Toll-like receptors on cells. All chemicals used were obtained from Sigma-Aldrich (St Louis, MO, USA). Cell-conditioned media were collected after 24 h and used for cytokine analyses. The remaining cells were fixed and then stored in phosphate-buffered saline (PBS) until staining was conducted (within 24 h of fixation).

### Phagocytosis Assay

Human adult microglia were plated at a density of 10,000 per well in 200 μl of the medium described above in flat-bottomed 96-well plates and left untreated for 48 h. The cells were then incubated for 48 h with LPS (100 ng/ml) or with the combination of LPS (100 ng/ml) and 10 μM dipyridamole (added 30 min before LPS). Ten microliters of yellow-green phagocytotic fluorospheres (Invitrogen, Carlsbad, CA, USA) were then added. After 2 h of incubation with the fluorospheres, the wells were washed twice with PBS, fixed in 4% paraformaldehyde for 10 min and then washed again with PBS. Rabbit anti-Iba1 (1 h at 1:100; Wako Chemicals USA, Richmond, VA, USA) and donkey anti-rabbit Texas Red (1 h at 1:200) were used to label the microglia. The Iba1 antibody labels ionized calcium-binding adapter molecule 1 that is selective to macrophage/microglia. Hoechst dye (10 min at 1:100) was used to label the nuclei. Images were acquired using the ImageXpress Micro XL Widefield High Content Screening System (Molecular Devices).

### Immunofluorescence cell labeling and quantification of image parameters

Fixed cells were stained for CD14 (1:40; BD Pharmingen), a cell surface LPS-binding coreceptor on monocytoid cells and secondary antibody before cell nuclei were labeled using Hoechst dye (1:100 for 10 min). The plate wells were then scanned automatically using the ImageXpress^MICRO^ controlled by MetaXpress hardware. Images were stored separately in TIFF file format as single-image planes for each wavelength. Image background was removed by using an image-flattening algorithm to permit between-well quantitative comparison. All image analysis was conducted using MATLAB and confirmed with test images, manual measurements and counting, and other software when available. Cell counts were based on colocalization of Hoechst dye with CD14. Cell areas and average cell intensities were measured by manually preselecting a threshold above local background for each 96-well plate, confirming appropriate thresholds by visual inspection of images and calculating average areas using standard MATLAB segmentation algorithms, morphological operators and measurement routines. Similar analyses were performed on images of spinal tissue stained for Iba1.

### ATP luminescence assay

Cell viability was assessed by use of an ATP assay kit according to the instructions of the manufacturer (CellTiter-Glo Luminescent Cell Viability Assay; Promega, Madison, WI, USA).

### Cytokine analyses

Cytokine concentrations within the microglia conditioned medium were measured with a single-cytokine ELISA and a multiplex ELISA. The TNF-α ELISA was done using a KHC3011 kit from Invitrogen. Conditioned media were also subjected to a multiplex human cytokine panel (LHC0009), permitting simultaneous measurement of 25 different cytokines and chemokines (Invitrogen).

### Disease induction and EAE analyses

EAE was induced in female C57BL/6 mice (The Jackson Laboratory, Bar Harbor, ME, USA), ages 8 to 9 wk, by subcutaneous (s.c.) injection of 50 μg MOG_35–55_ in Freund’s Complete Adjuvant medium (Thermo Fisher Scientific, Rockford, IL, USA) on day 0. Intraperitoneal (i.p.) pertussis toxin (0.1 μg/200 μl; List Biological Laboratories, Hornby, ON, Canada) was administered on days 0 and 2. To increase the sensitivity of measurement, animals were assessed daily using a 15-point disease score scale [27,51] instead of the more commonly used 5-point scale. The 15-point scale differentiates individual limb disability rather than grouping both fore- or hindlimbs together. The 15-point scale (0 to 15) is the sum of the disease state for the tail (scored from 0 to 2) and each limb (scored from 0 to 3), and death is scored at 15. All animals were handled in accordance with the policies outlined by the Canadian Council for Animal Care and the University of Calgary.

Dipyridamole was suspended in 0.5% carboxymethylcellulose and, unless otherwise stated, was given to mice through oral gavage daily beginning on day 7 in 100 μl of suspension at a final dose of 100, 200 or 300 mg/kg. These doses were chosen because the uppermost safety limit for dipyridamole in mice is 400 mg/kg (lethal dose 50, or LD50, is 700 mg/kg) [52].

### Histology of tissue from control and EAE mice

Animals were killed by giving them an overdose of ketamine/xylazine (200 and 10 mg/kg, respectively). Spinal cords were removed and dissected into preselected parts to maximize opportunities for histological analyses, and the thoracic blocks (1.0 cm) were immersion-fixed in 10% buffered formalin and then embedded in paraffin. Thoracic sections were cut on a microtome at 6-μm thickness and mounted on glass slides. For each mouse, the thoracic cord was cut longitudinally through the entire dorsal–ventral axes. Six sequential series of sections, spaced 50 μm apart, were generated. One series was processed for histological staining with hematoxylin-eosin and Luxol fast blue as previously described [27,51], and another was processed for Iba1. We focused our attention on the T3/T4 segments.

Images of the sections were captured using an Olympus BH2 microscope (Olympus America, Center Valley, PA, USA) and QCapture Pro version 5.1.1.14 software (QImaging, Surrey, BC, Canada). For semiquantitative assessment of the extent of inflammation and demyelination in the spinal cord, all stained longitudinal sections from the same coded mouse were evaluated using a scoring system described previously [27]. In this regard, the location (pia vs parenchyma) and number of inflammatory aggregates per section were documented, with parenchymal inflammation and larger and higher number of aggregates being assigned greater inflammation scores. The reader is referred elsewhere for the details [27]. Six sections per spine were evaluated by a blinded observer, and the average score per mouse was documented.

### Statistical analysis

Statistical analysis was performed using R version 2.8.1 software (The R Foundation for Statistical Computing, Vienna, Austria) and MATLAB version 7.7 software. Statistical differences for cells in culture (Figures 1, 2 and 3) were addressed using ANOVA with the Bonferroni correction for multiple comparisons. Statistical differences between groups of mice in the EAE experiments (Figures 4, 5 and 6) were evaluated using a nonparametric Mann-Whitney *U* test. An α score of 0.05 was selected for statistical significance.

## Competing interests

The authors have no competing interests to declare.

## Authors’ contributions

SS planned the entire study, performed the tissue culture and EAE experiments, evaluated the histopathology, analyzed the data and wrote the first draft of the manuscript. WH provided the surgical specimens and edited the manuscript. YS provided the surgical specimens and edited the manuscript. LM helped cosupervise the study and edited the manuscript. VWY coplanned the entire study, provided overall supervision and finalized the manuscript. All authors read and approved the final manuscript.
